# Listeners’ Linguistic Experience Affects the Degree of Perceived Nativeness of First Language Pronunciation

**DOI:** 10.3389/fpsyg.2021.717615

**Published:** 2021-10-08

**Authors:** Lisa Kornder, Ineke Mennen

**Affiliations:** Department of English Studies, University of Graz, Graz, Austria

**Keywords:** first language attrition, bilingualism, foreign accent, nativeness perception, English, (Austrian) German

## Abstract

The aim of this study was to explore if and to what extent Austrian-English late sequential bilinguals who have been living in a second language (L2) environment for several decades are perceived to sound native in their first language (L1) when being compared to monolingual Austrian German (AG) control speakers. Furthermore, this investigation aimed to identify if listeners differ in their judgments of nativeness of L1 pronunciation depending on their own language background. For this purpose, two groups of native Austrian German listeners (*N* = 30 each), who differed regarding their linguistic background (Austrian German monolingual and Austrian German-English bilingual listeners) were asked to rate spontaneous speech samples produced by Austrian English bilingual and Austrian German monolingual speakers. Results showed that the bilingual L1 speech was perceived to sound overall less native compared to monolingual control speech. It was further observed that the two listener groups significantly differed in their perception of nativeness: Bilingual listeners were overall less likely to judge bilingual L1 pronunciation to sound non-native compared to monolingual listeners. To date, this is the first study to show that listener experience influences their perception of nativeness of L1 pronunciation and, thus, adds a new dimension to the notion of the native speaker.

## Introduction

A speaker’s accent, shaped by various segmental and prosodic characteristics, is one of the most salient features of speech production and communication. Research shows that listeners are very sensitive to accented speech (e.g., Flege, 1984; Munro and Derwing, 1995b; Magen, 1998; Scales et al., 2006; Major, 2007; Eger and Reinisch, 2019) and that their perception and judgment of an individual is strongly influenced by the speaker’s pronunciation (e.g., Lindemann, 2002; Kang and Rubin, 2009; Kang et al., 2016). In second language (L2) acquisition research, considerable attention has been given to the examination of features related to L2 learners’ and bilinguals’ accent, resulting from the observation that individuals who acquire an L2 relatively late in life often retain influences of their first language (L1) in their L2 pronunciation (Scovel, 1969; Flege, 1980, 1981; Flege et al., 1996; MacKay et al., 2001). This has led to acoustic investigations of the extent to which influences from the L1 lead to segmental and prosodic divergences from L2 production norms (e.g., Flege, 1991; Thornburgh and Ryalls, 1998; Flege et al., 2003; Baker and Trofimovich, 2005; Simon, 2009; Levy and Law, 2010), as well as numerous studies that examine the perception of non-native L2 speech by (predominantly) monolingual listeners (e.g., Bongaerts et al., 1995; Flege et al., 1995; Munro and Derwing, 1995a; Riney and Flege, 1998; Moyer, 1999; Jilka, 2000; Kang et al., 2016).

While it is well-established that a speaker’s L2 accent is likely to show traces of the L1 system, more recent research shows that the reverse is also possible, that is, a late-acquired L2 might affect a speaker’s L1 accent due to bidirectional interaction processes taking place between the L1 and the L2 system (e.g., Mennen, 2004; Dmitrieva et al., 2010; Mayr et al., 2012; Bergmann et al., 2016; de Leeuw et al., 2017; Stoehr et al., 2017; de Leeuw, 2019). This phenomenon, referred to as *L1 attrition*, is frequently observed among bilinguals who have been long-term immersed in an L2 speaking country and who acquired their L2 after adolescence, i.e., at a point when the L1 is already fully developed in healthy individuals (Köpke and Schmid, 2004). Attrition research has provided evidence for the malleability of the L1 system with regard to segmental (e.g., de Leeuw et al., 2012a; Mayr et al., 2012; Stoehr et al., 2017; de Leeuw, 2019; Kornder and Mennen, 2021) and prosodic (de Leeuw et al., 2012b; Mennen and Chousi, 2018; de Leeuw, 2019; Gargiulo and Tronnier, 2020) features in L2-immersed late sequential bilinguals with different language backgrounds. These studies show that phonetic and phonological features of the L1 system might shift toward L2 production norms as a result of L2 learning experience and long-term exposure to the L2. The extent to which such L2-induced modifications in a bilingual’s L1 accent are discernible for listeners has only recently started to attract attention (Sancier and Fowler, 1997; Hopp and Schmid, 2013; Schmid and Hopp, 2014; Bergmann et al., 2016; Mayr et al., 2020). Findings of accent rating studies show that listeners are indeed sensitive to divergences from L1 pronunciation patterns in bilingual speakers, which suggests that changes in L1 pronunciation resulting from long-term exposure to an L2 might lead to a detectable non-native L1 accent (e.g., De Leeuw et al., 2010; Bergmann et al., 2016; Mayr et al., 2020).

Following this line of inquiry, the present study aimed to contribute to the research on listener perceptions of potentially attrited L1 speech by conducting L1 nativeness ratings in a group of Austrian German-English late sequential bilinguals and monolingual Austrian German control speakers. In addition, this study set out to explore if and to what extent the linguistic background of listeners, that is, whether they are (quasi-)monolingual speakers of Austrian German or German-English bilingual speakers, affects their judgment of nativeness in L1 Austrian German pronunciation. In this context, we also examined if the extent to which bilinguals are perceived to sound (non-)native in their L1 pronunciation changes at different stages after immigrating to an L2 speaking country, judged by the rater groups described above. The decision to include two rater groups differing in terms of their language background is based on recent findings concerning L2 phonetic and phonological influences on L1 perception, showing that bilinguals’ L1 perceptive abilities might be modified as a result of L2 learning experience (e.g., Celata and Cancila, 2010; Major, 2010; Carlson, 2018; Cabrelli et al., 2019). Hence, the present study sought to determine if differences in listeners’ linguistic background may lead to differences in their perception of L1 nativeness.

### First Language Phonetic Attrition

First language phonetic attrition refers to non-pathological modifications of L1 phonetic features resulting from long-term L2 learning experience and L2 exposure in bilinguals who acquired their L2 relatively late in life and who have been immersed in a migration setting for an extended period of time (e.g., Seliger and Vago, 1991; Köpke and Schmid, 2004). It should be noted though that L2-induced changes in the L1 system are not restricted to highly experienced bilinguals who have been permanently living in an L2 setting for a considerable period of time (see Kartushina et al., 2016a, for an overview). Bi-directional interaction processes between a speaker’s linguistic systems leading to a shift of phonetic categories in the direction of the L2 might already occur at an early stage of L2 learning (e.g., Chang, 2012, 2013), as a result of recent and focused L2 production training (Dmitrieva et al., 2010; Kartushina et al., 2016b), or as a result of traveling between an L1 and an L2 country on a regular basis (Sancier and Fowler, 1997; Tobin et al., 2017). In these contexts, changes in the L1 pronunciation system are not indicative of L1 attrition, but represent instances of *phonetic drift* (Chang, 2012) of L1 categories toward L2 categories, that is, rather subtle L1 changes resulting from recent and increased L2 input. Phonetic drift, as described by Chang (2012, p. 264), can be considered as “one step in a continuum of cross-linguistic effects in bilinguals dependent on relative use of the L1 vs. the L2.” Such subtle L2-induced modifications of the L1 pronunciation system occurring at an early stage of L2 learning have been shown to revert back to native L1 norms when speakers experience changes in their linguistic environment through, for instance, moving back to their L1 environment, and changes in language use (e.g., Chang, 2019). As such, instances of phonetic drift are not considered being indicative of L1 attrition given that they do not represent a decline in L1 proficiency (see e.g., Chang, 2012, 2013, 2019; Kartushina et al., 2016b). The present investigation focuses on potential L1 attriters, that is, late sequential bilinguals who are experienced L2 speakers and who have been living in an L2-speaking country for several decades, in which more persistent changes in L1 speech are reported to occur (e.g., Mayr et al., 2012; de Leeuw, 2019; Kornder and Mennen, 2021).

Empirical findings from research on L1 phonetic attrition provide evidence that a mature L1 pronunciation system is sensitive to L2 influences—in the same way a late-acquired L2 system is likely to be influenced by the native pronunciation system (e.g., Flege, 1980, 1981; Flege et al., 1996; Piske et al., 2001). These findings are in line with the Speech Learning Model (SLM) (Flege, 1995; Flege and Bohn, 2021), a model of L2 pronunciation development which predicts bidirectional influences between the L1 and L2 sound system, that is, the L1 influences the L2 system and vice versa. L1 segmental modifications in the direction of L2 norms have been predominantly investigated with regard to bilinguals’ productions of plosive consonants (Flege, 1987a; Mayr et al., 2012; Stoehr et al., 2017; Kornder and Mennen, 2021) and vowels (Mayr et al., 2012; Bergmann et al., 2016; de Leeuw, 2019; Kornder and Mennen, 2021). Other segmental features which have been shown to undergo attrition include, for instance, the post-vocalic rhotic consonant/r/ (Ulbrich and Ordin, 2014) and the lateral approximant/l/ (de Leeuw et al., 2012a; de Leeuw, 2019). By contrast, the extent to which L1 prosodic features are likely to be affected by L2-induced changes has been less frequently examined so far (de Leeuw et al., 2012b; Mennen and Chousi, 2018; de Leeuw, 2019), but findings suggest that also intonation features, such as tonal alignment (de Leeuw et al., 2012b; Mennen and Chousi, 2018), might shift in the direction of the L2.

The extent to which segmental and prosodic divergences from L1 norms lead to a perceived global foreign accent in L2-immersed late sequential bilinguals’ L1 pronunciation has been explored in a series of foreign accent rating studies (FARs) (Sancier and Fowler, 1997; De Leeuw et al., 2010; Hopp and Schmid, 2013; Schmid and Hopp, 2014; Bergmann et al., 2016; Mayr et al., 2020). Overall, the findings of these studies confirm that L2-induced modifications in a speaker’s L1 pronunciation system are in many cases distinctively discernible for listeners. De Leeuw et al. (2010), for example, examined the degree of perceived foreign accent in a group of German-English and German-Dutch bilinguals living in an L2 environment. Their findings revealed that monolingual German listeners were more likely to judge the L1 pronunciation of the bilingual group as sounding foreign compared to the speech of monolingual German controls. Similar observations were made by Bergmann et al. (2016) who found that nearly 40% of their German-English bilinguals received significantly higher foreign accent ratings compared to monolingual control speakers, that is, monolingual German listeners perceived some of the bilinguals to sound clearly non-native in their L1 German while others scored within the native range. The observation that not necessarily all bilinguals are perceived to have a non-native L1 accent was also made by Hopp and Schmid (2013), who showed that monolingual German listeners judged the majority of German-English and German-Dutch bilinguals (*N* = 29 out of 40) to sound not significantly differently from German control speakers in their L1 German pronunciation. At the same time, the ratings obtained for the remaining bilinguals (*N* = 11 out of 40) were clearly below the native German range.

While the accent rating studies outlined above focused on bilinguals with L1 German, Mayr et al. (2020) assessed perceived nativeness in monolingual Spanish speakers and two groups of native Spanish speakers of English. One group included Spanish-English bilinguals who taught Spanish as an L2 in the United Kingdom (teachers), the other group consisted of non-teachers with L1 Spanish who also lived in the United Kingdom and were late learners of L2 English. FAR results showed that monolingual Spanish listeners attributed overall higher accent scores to the L1 Spanish produced by the teachers compared to the non-teachers and monolingual Spanish controls. Mayr et al. (2020) argued that this difference might be explained by the fact that the teachers were immersed in professional environments with a high level of language co-activation and regular exposure to learners’ non-native pronunciations. Similar observations were made by De Leeuw et al. (2010) who found that L1 accent ratings were overall highest for native German speakers of L2 English or L2 Dutch who were predominantly immersed in communicative settings in which code-mixing was enhanced, leading to higher levels of language co-activation. By contrast, monolingual German controls and German-English/Dutch bilinguals who were immersed in settings in which code-mixing was less likely to occur received comparatively lower accent ratings.

Another study frequently discussed in the context of perceived L1 accent was conducted by Sancier and Fowler (1997) who asked monolingual English and monolingual Brazilian-Portuguese listeners to rate speech samples produced by a Portuguese-English late bilingual. They aimed to identify potential changes in the speaker’s L1 Portuguese and L2 English accent after staying in an L1 (Brazil) and L2 (United States) environment for several months at a time. While native English listeners did not perceive the speaker’s L2 accent to be significantly different after staying in the United States and Brazil, respectively, native Brazilian-Portuguese listeners perceived the speaker’s L1 pronunciation to be more accented after being exposed to English for an extended period of time. While this study is often cited in the context of L1 attrition research, it should be noted that the subject examined by Sancier and Fowler is—based on the definition of L1 phonetic attrition outlined above—not an attriter in the strictest sense given that she experienced regular changes in her linguistic environment. Nevertheless, the findings presented in this study do not only show that a bilingual’s L1 accent is sensitive to recent and enhanced L2 input, but that the resulting pronunciation changes are also detectable for listeners.

Overall, the findings outlined above confirm that L2-induced phonetic and phonological modifications in a speaker’s L1 system often lead to a non-native accent in the L1. Accent rating studies offer valuable insights into pronunciation differences between monolingual and bilingual speakers and show that listeners are sensitive to diverging L1 pronunciation patterns. Furthermore, they reveal that the extent to which L2-immersed bilinguals are perceived to have a foreign accent in their L1 varies, that is, bilingual speakers are not inevitably perceived to sound less native compared to monolingual controls (De Leeuw et al., 2010; Hopp and Schmid, 2013; Bergmann et al., 2016). There are several possible reasons for this: First, a bilingual’s L1 pronunciation system is not necessarily affected by attrition, that is, some speakers will not show signs of phonetic attrition (e.g., Bergmann et al., 2016) or attrition in other linguistic areas. Hence, it is unlikely that we would perceive them as different from monolingual speakers. Second, not all acoustic changes a speaker’s L1 system might undergo will be perceived as non-native or foreign by listeners. Research has shown that modification processes influence bilinguals’ L1 system selectively rather than causing changes in the entire L1 system, which may result in both more pronounced changes on the one hand, and relatively subtle modifications on the other hand (e.g., Mayr et al., 2012; Bergmann et al., 2016; Kornder and Mennen, 2021). Some of these changes might be too subtle to be perceived by listeners or—even if they are perceived—they may not necessarily be rated as non-native. Furthermore, research suggests that perceiving an individual’s pronunciation as non-native is not based on a single acoustic-phonetic feature, but rather results from an interplay between and accumulation of different segmental and prosodic features which diverge from expected pronunciation patterns (e.g., Jilka, 2000; Mennen, 2004; Bergmann et al., 2016; Ulbrich and Mennen, 2016; van Maastricht et al., 2017). Therefore, even if acoustic-phonetic analyses reveal modifications in specific L1 segmental and/or prosodic features, they may only lead to a perceived non-native accent in combination with other phonetic and phonological changes (e.g., Bergmann et al., 2016).

The third reason for the observation that not all bilinguals are perceived to sound (non-)native to the same degree relates to listener-dependent variables which might affect their perception of accented speech, including their own language background, i.e., are they monolingual or bilingual speakers, and their familiarity with non-native or accented speech, i.e., are they exposed to non-native speech on a regular basis. The role of listener variables has predominantly been explored in the context of perceived accentedness of L2 speech (e.g., Thompson, 1991; Flege and Fletcher, 1992; Kennedy and Trofimovich, 2008; Eger and Reinisch, 2019), but recent research suggests that rater effects, such as their familiarity with the target language, may also affect the perception of L1 speech (Schmid and Hopp, 2014). There is to date no empirical research which set out to systematically examine how listeners’ linguistic background and experience with an L2 influences their perception of L1 speech produced by monolingual and bilingual speakers—a gap the present investigation aims to fill, as will be further outlined below.

### Listener Variables

As pointed out above, differences in foreign accent ratings are likely to result from both speaker-specific and listener-specific features, with the latter being predominantly investigated in L2 acquisition research. Thompson (1991), for instance, identified a significant relationship between listeners’ linguistic experience and perceived nativeness of L2 English speech produced by L1 Russian speakers. Native English listeners were considered experienced if they were fluent in any additional language and had frequent contact with non-native English speakers. The L2 accent rating results showed that experienced listeners were less likely to rate English speech samples produced by L2 English subjects as non-native than inexperienced English raters who did not speak an L2 and who were not exposed to non-native English speech on a regular basis (see also Cunningham-Andersson, 1997). These findings corroborate Long’s (1990) observation that listeners who have experience with other languages and live, for instance, in linguistically diverse communities are likely to be more lenient toward non-native accents compared to listeners who lack linguistic experience. Eger and Reinisch (2019) also showed a correlation between listener experience and foreign accent ratings, which, however, points in the opposite direction. They examined the extent to which native German learners’ proficiency in L2 English affects their perception of German-accented English speech. Similar to the present study (see section “Speakers and Speech Samples,” for more details), experience was defined as listeners’ overall L2 proficiency, based on their self-reported skills in and frequency of speaking and listening in English. In Eger and Reinisch’s (2019) study, listeners were asked to rate the “goodness” of the pronunciation of individual English target words produced by German learners of English. Their findings suggest that the more proficient listeners were in their L2, the less likely they were to accept German-accented productions as “good” representations of the L2 target, that is, an increase in L2 proficiency was correlated with a higher sensitivity to accented L2 speech.

Other studies examining the perception of L2-accented speech, by contrast, do not show a correlation between listeners’ linguistic experience and accent ratings. Kennedy and Trofimovich (2008), for example, investigated the extent to which listeners’ experience with Mandarin-accented English influences their perception of foreign accentedness, intelligibility and comprehensibility. While the findings showed that more experienced listeners perceived non-native English samples to be more intelligible, experience was not observed to have an impact on listeners’ ratings of accentedness. Similarly, Flege and Fletcher (1992) did not find a significant relationship between linguistic experience, defined as listeners’ familiarity with non-native speech, and perceived accentedness. Also Major (2007) found that listeners’ familiarity with the language to be rated did not have a significant impact on their perception of (non-)native speech, that is, listeners were equally able to distinguish between native and non-native Brazilian-Portuguese speech samples.

The extent to which listeners’ linguistic background and experience play a role in the perception of accented L1 speech has not been investigated in greater detail so far. The only study—to the best of our knowledge—which addresses the impact of listener-specific variables on the perception of not only non-native L2 speech but also on bilinguals’ L1 pronunciation was conducted by Schmid and Hopp (2014) who examined how variation in FAR scores can be attributed to rater differences, among other variables. Their study involved three groups of speakers, namely monolingual German controls, L1 German speakers of L2 English or L2 Dutch (=L1 German attriters), and L1 English or L1 Dutch learners of German (=L2 German learners). Both native and non-native German raters who studied L2 English at university level in Germany were asked to rate German speech samples produced by the different speaker groups according to perceived foreign accent. Findings showed a correlation between raters’ familiarity and contact with the target language German and foreign accent scores obtained for the monolingual German speaker group. That is, raters who were less familiar with German were more likely to judge monolingual German speech samples as non-native. No such correlations were demonstrated for the ratings obtained for the bilingual speech samples (i.e., L1 German attriters and L2 German learners), suggesting that listeners’ familiarity and contact with a specific language only influence their perception of monolingual speech while their perception of potentially attrited and L2 speech remains largely unaffected. Based on these findings, Schmid and Hopp (2014, p. 383) conclude that “[v]ariation in raters can lead to shifts in the absolute assessment of the strength of foreign accents on a given scale, with some raters apparently being more strict in the threshold of whom they judge to be native or native-like”.

### Objectives

As previously outlined, early linguistic research focusing on bilingualism and L2 acquisition was conducted under the premise that the L1 is protected against influences from a late-acquired L2 due to biological maturation processes (Lenneberg, 1967; Scovel, 1969). That is, a fully developed and mature L1 system was not considered to be modified or become less accessible in response to the acquisition of an additional language. Empirical investigations, however, questioned the stability of the L1 system in that they revealed that L2 learning experience can, in fact, lead to changes in a speaker’s L1 system (e.g., Flege and Hillenbrand, 1984; Flege, 1987b; Major, 1992; Schmid, 2002; Mennen, 2004; Mayr et al., 2012; de Leeuw et al., 2017). From the perspective of a dynamic systems approach to language development, temporal or permanent modifications of a speaker’s linguistic systems—including both the second and the L1—are regarded as an inherent part of language acquisition and development, resulting from dynamic, ongoing L1–L2 interactions (e.g., de Bot, 2007; de Bot et al., 2007). Hence, studies such as the present one contribute to arriving at a more profound understanding of the complexity and dynamics of developmental processes involved in language acquisition and of what might happen to a speaker’s L1 pronunciation system when an additional language is acquired. The flexibility and malleability of a mature L1 system has attracted increasing attention among linguists and L2 researchers in the past decades, as the growing number of studies exploring the phenomenon of L1 attrition shows. These include empirical investigations of L1 attrition in all linguistic areas, such as syntax (e.g., Schmid, 2002; Tsimpli et al., 2004), the lexicon (e.g., Ammerlaan, 1996; Schmid and Jarvis, 2014), morphology (e.g., Altenberg, 1991), semantics (e.g., Jaspaert and Kroon, 1992; Pavlenko and Malt, 2011), and phonetics and phonology (e.g., Mayr et al., 2012; Bergmann et al., 2016; Cho and Lee, 2016; de Leeuw et al., 2017; Mennen and Chousi, 2018; de Leeuw, 2019). The development toward acknowledging that a bilingual’s linguistic configuration is characterized by mutual L1–L2 interactions confirms Grosjean (1989, p. 13) former prediction that linguists “will no longer examine one of the bilingual’s languages without examining the other,” which gave rise to an integrated and holistic view on bilingualism (see also Grosjean, 1997). Moreover, the possibility that bilinguals might end up speaking not only their L2 but also their L1 with a detectable non-native accent is relevant from a sociolinguistic point of view, questioning the idealized image of the native speaker and pointing to the necessity to reconsider the native speaker norm and its relevance in linguistic research (see Davies, 2003). When it comes to the question of who is considered to be a native speaker and how nativeness is assessed, examining effects of listeners’ language background and experience on their own perception of L1 speech is crucial. Not only do the findings of such investigations entail methodological consequences for the design of accent rating studies (see Schmid and Hopp, 2014, for a discussion), but they also add yet another dimension to the discussion of whether the native speaker concept in its traditional definition is still maintainable.

Based on the above considerations, the present study aimed to contribute to the emerging body of research exploring listener perceptions of potentially attrited L1 speech in order to determine if and to what extent L2-immersed bilinguals are perceived to have a non-native accent in their L1 when being compared to monolingual speakers (De Leeuw et al., 2010; Hopp and Schmid, 2013; Schmid and Hopp, 2014; Bergmann et al., 2016; Mayr et al., 2020). In addition, this study sought to investigate if listeners’ language background and linguistic experience (monolingual vs. bilingual listeners) affect their perception of L1 pronunciation, that is, to find out whether there are any significant differences between monolingual and bilingual listeners in terms of their perception of nativeness in L1 speech. To this end, two groups of phonetically untrained listeners, (quasi-)monolingual Austrian German and bilingual AG-English listeners from Austria, were invited to rate a set of spontaneous L1 speech samples produced by AG-English late sequential bilinguals and monolingual AG controls according to perceived nativeness of pronunciation.

## Materials and Methods

### Speakers and Speech Samples

Speech samples from AG-English bilinguals and AG monolinguals were extracted from publicly available German TV and radio interviews (see Supplementary Table 1). The bilingual group included three male AG-English celebrities, who are long-term United States immigrants and started acquiring English as an L2 in early adulthood. The first bilingual, Arnold Schwarzenegger (AS), was born in Thal, Styria (Austria), in 1947 and moved to the United States at the age of 21, where he made a career in bodybuilding, acting and politics (Schwarzenegger, 2012). Having learned English as a foreign language in an instructional setting in Austria, Schwarzenegger had only moderate English skills when he migrated to the United States (Outland Baker, 2006). Similar to Schwarzenegger, Frank Stronach (FS), who was born in a small municipality in East Styria (Austria) in 1932, left his home country in early adulthood and migrated to Canada in 1954 (Mayr, 2013; Noble, 2014). There, he started his first business which laid the foundation for a successful entrepreneur career. The third bilingual, Wolfgang Puck (WP), is an Austrian-born celebrity chef who moved from St. Veit an der Glan (Austria) to Los Angeles in 1973 where he started learning English (Schoenfeld, 2003).

The control group included five monolingual AG male speakers, aged 69–78, who were born and raised in Thal (Austria). Unlike the bilingual subjects, the control speakers were not well-known public figures, but locals who had been informally interviewed on different occasions in Thal. Given that the bilingual samples represented non-prompted, semi-spontaneous speech, the control samples were also selected from pre-recorded broadcast interviews, i.e., all speech samples included in the rating task were produced in a non-experimental setting. While the majority of accent rating studies rely on rehearsed (e.g., Elliott, 1995; Derwing and Munro, 1997) or read (e.g., Thompson, 1991; Moyer, 1999) speech, this is, to our knowledge, the first L1 nativeness perception study which had listeners rate instances of non-prompted spontaneous speech. Using non-prompted speech samples can be considered being more representative of a speaker’s natural and authentic pronunciation than speech samples elicited in a strictly controlled experimental setting (see e.g., Long, 1990).

From the broadcast interviews, bilingual and control samples were selected following a set of pre-defined criteria (see Jesney, 2004; Schmid and Hopp, 2014, for discussions). In order to ensure that listeners base their nativeness judgments on pronunciation-related features only, the samples did not include lexical and grammatical errors or hesitation and disfluency markers (e.g., Lennon, 1990). In addition, speech samples containing code-switches, high levels of background noise, longer pauses or self-corrections were excluded. Based on these criteria, a total of 28 speech samples was included in the speech corpus. For speaker AS, two different sets of samples were selected, one representing his early pronunciation in the late 1970s, and the other one representing his more current, late pronunciation in the 2010s. By comparing the ratings assigned to his early and late speech, we were able to explore if his late pronunciation was rated differently in terms of perceived nativeness compared to his early pronunciation. No such comparison could be drawn for the bilinguals FS and WP given that usable audio recordings representing their pronunciation at an earlier stage of migration were not available at the time the present study was conducted. Using speech samples produced by a single subject may constitute a limiting factor in our study, but the findings obtained in this single-subject investigation have the potential to serve as an incentive for future large-scale studies to explore if speakers’ L1 pronunciation is likely to be perceived more or less native in the course of L2-immersion.

The individual samples varied in total duration, ranging from 1.95 to 5.39 s (*M* = 3.78, *SD* = 0.91; see Table 1). Previous L1 accent rating studies made use of considerably longer stretches of speech, in the range of approximately 10–20 s (e.g., Hopp and Schmid, 2013; Bergmann et al., 2016; Mayr et al., 2020). In the present study, however, shorter durations were selected to reduce the possibility that listeners identify the speakers, in particular the three bilinguals who, as mentioned above, are well-known celebrities. The length of the individual speech samples was considered sufficient for listeners to make their judgments given that we know from previous research that listeners are well able to rate speech samples which are very short in duration (see Flege, 1984). Empirical studies also show that listeners’ ability to recognize speakers they are already familiar with is influenced by the duration of the speech samples they are presented with. Not surprisingly, speech samples which are longer in duration increase the likelihood that listeners identify a speaker (e.g., Schweinberger et al., 2013; see Mathias and von Kriegstein, 2014, for a discussion). However, research also indicates that listeners are well able to identify familiar speakers in speech samples which are shorter than 500 ms (see e.g., Fontaine et al., 2017). It has to be noted though that in these studies, listeners were specifically tested on their ability to recognize speakers based on voice recordings, i.e., listeners can be assumed to have made a special effort to identify the speaker, and were presented with a closed set of speakers to choose from. Recognition rates are reported to drop to above chance when identifying celebrities from an open response set (Van Lancker et al., 1984, 1985). In the present study, by contrast, the task was *per se* different in that listeners were not asked to pay attention to the speakers’ identity, but rate their pronunciation according to perceived nativeness. After completing the rating task, listeners were asked on the rating sheet if they had noticed something about one or more of the speakers. Given that we did not want listeners to make a conscious effort to identify the speakers they were listening to, we did not specifically ask them *Did you recognize one or more of the speakers?*, but formulated a more open question, which still gave listeners the opportunity to mention if they had identified a speaker. In fact, *N* = 8 listeners, who were originally asked to complete the rating task, answered this question by stating that they had recognized speaker AS and/or speaker FS in some (but not all) of the speech samples. These listeners were excluded from analysis. In order to further ensure that listeners would not be able to detect who is speaking, the selected speech samples did not contain place and proper names which may uncover the identity of the speaker.

**TABLE 1:** Overview of speakers and speech samples.

	**N subjects**	**N samples**	**Mean word count (SD)**	**Mean duration in sec. (SD)**
Bilinguals	3	18	9.3 (2.06)	3.58 (0.88)
Controls	5	10	12 (3.27)	4.15 (0.89)

	**N_total_ subjects**	**N_total_ samples**	**Total word count**	**Total duration (sec.)**

	8	28	288	105.91

### Listeners

A total of 60 listeners was recruited at the Department of English Studies at the University of Graz (Austria) and *via* personal contacts in Graz. Depending on their language background and English proficiency, the subjects were assigned to two different groups (*N* = 30 each). Monolingual (ML) raters (13 male, 17 female),^1^ aged between 23 and 43 (*M* = 32.67, *SD* = 5.23), were (quasi-)monolingual^2^ speakers of Austrian German, who reported having learned English in school for 5–9 years (*M* = 6.97, *SD* = 1.19), but rarely or never actively used English in private or professional contexts (see Table 2). Bilingual (BIL) raters (14 male, 16 female), aged between 22 and 27 (*M* = 24.1, *SD* = 1.32), were Austrian German learners of L2 English who were undergraduate students of English and American Studies at the University of Graz in their 2nd to 4th year (*M* = 3.05, *SD* = 0.67). Subjects in this group reported using English not only at university, but making moderate to frequent use of English in different communicative contexts outside university (see Table 2). Raters in both groups were born and raised in a monolingual AG environment in Graz and were permanent residents of Graz or surrounding areas. All participants reported normal hearing.

**TABLE 2:** Listeners’ self-reported frequency of English use in different contexts (1 = *Never*, 2 = *Very rarely*, 3 = *Rarely*, 4 = *Frequently*, and 5 = *Very frequently*).

	**ML (*N* = 30)**			**BIL (*N* = 30)**		
	***Median***	***Mean (SD)***	***Min-max***	***Median***	***Mean (SD)***	***Min-max***
Speaking (private)	*	*	*	3.5	3.5 (1.04)	1.0–5.0
Speaking (professional)	3.0	2.37 (0.99)	1.0–4.0	3.0	2.39 (1.38)	1.0–5.0
Watching (TV/films)	2.0	2.03 (1.07)	1.0–4.0	4.0	4.47 (0.51)	4.0–5.0
Reading	1.0	1.5 (0.73)	1.0–3.0	4.0	3.77 (0.68)	3.0–5.0
Listening	1.0	1.07 (0.25)	1.0–2.0	3.0	3.0 (1.34)	1.0–5.0
Writing	1.0	1.37 (0.72)	1.0–3.0	3.0	2.77 (1.45)	1.0–5.0

*All ML listeners reported that they never used English in private contexts. ML, monolingual listeners; BIL, bilingual listeners.

In order to obtain information concerning raters’ linguistic background and language use, each subject was invited to fill in a questionnaire. Participants were asked to self-assess their overall English competence based on the six competence levels defined by the Common European Framework of Reference for Languages (Council of Europe, 2001), which resulted in a six-point Likert scale ranging from 1 = very basic user to 6 = native or near-native user. ML listeners’ self-assessed English proficiency ranged from 1 to 4 (*M* = 2.6, *SD* = 0.89) while the BIL listeners rated their proficiency in English from 4 to 6 (*M* = 4.7, *SD* = 0.53). Results of a Mann-Whitney *U* test showed that the two listener groups significantly differed in their self-assessed English proficiency (*U* = 870, *r* = 0.829, *p* < 0.001). In addition, each subject had to indicate if (*yes*/*no*) and how frequently (*very rarely*/*rarely*/*frequently*/*very frequently*) they used English across the four skills, i.e., speaking (professional vs. private), listening, reading, and writing. Their answers were converted to a five-point scale, ranging from 1 = never to 5 = very frequently. As shown in Table 2, participants in the BIL group used English overall more frequently in different contexts compared to the ML subjects, who used English either not at all or with low frequency only. Mann-Whitney *U* tests showed that the between-group differences in frequency of English use were significant for *Speaking (private)* (*U* = 870, *r* = 0.877, *p* < 0.001), *Watching* (*U* = 876, *r* = 0.838, *p* < 0.001), *Reading* (*U* = 878, *r* = 0.844, *p* < 0.001), *Listening* (*U* = 814, *r* = 0.764, *p* < 0.001), and *Writing* (*U* = 699, *r* = 0.52, *p* < 0.001). By contrast, the two groups did not differ in terms of frequency of English use in professional contexts (*p* = 1).^3^

Furthermore, the questionnaire collected information regarding subjects’ familiarity with Austrian German varieties in order to avoid that they misperceive an Austrian German Styrian regional variety as a non-native accent (e.g., Flege et al., 1997). Participants in both groups reported being familiar with different Austrian German varieties, in particular with the Styrian variety spoken in Graz and surrounding areas.

### Experimental Procedure

Due to the Covid-19-related restrictions in Austria, it was not possible to conduct the rating experiment in person. Therefore, rating materials and task descriptions were sent to the participants *via* e-mail. Each participant received a Power Point file, including the 28 test samples with one sound sample per slide, and detailed instructions. The rating sheet and the language background questionnaire were presented in an Excel file on two different spread sheets. As reported by some participants, it took approximately 15–20 min to complete the rating task.

The nativeness judgments were based on scalar ratings which have been used in previous accent rating studies to examine both perceived L2 (e.g., Moyer, 1999) and L1 (e.g., De Leeuw et al., 2010; Bergmann et al., 2016) accent. Raters were instructed to listen to one speech sample at a time and then (1) state whether the speaker is a native speaker of German (*yes*/*no*), and (2) indicate how certain they are concerning their judgment (*certain*/*relatively certain*/*uncertain*).^4^ In the analysis process, listener answers were transferred to a six-point Likert scale, with 6 = certain of non-native speaker status, 5 = semi-certain of non-native speaker status, 4 = uncertain of non-native speaker status, 3 = uncertain of native speaker status, 2 = semi-certain of native speaker status, and 1 = certain of native speaker status. Resulting from this, speakers who received a low rating score were perceived to sound native or near-native in their German pronunciation while speakers with a high rating score were perceived as non-native or near non-native speakers of German. The nativeness rating resulted in 1,680 individual rating scores (60 listeners × 28 ratings), which were averaged for the two rater groups (BIL vs. ML), for both speaker groups (bilinguals vs. controls) and for each of the bilinguals individually.

### Statistical Analysis

In order to examine if the two speaker groups differed in the nativeness ratings they obtained and if the two listener groups differed in their perception of nativeness of bilingual and monolingual L1 speech, we ran a series of two-way repeated ordinal regression analyses in R (R Core Team, 2020), using the *Anova.clmm* function from the R package *RVAideMemoire* (Hervé, 2020) to determine main and interactions effects, including *rating score* as the dependent variable in each model. *Post hoc* Tukey’s tests were conducted using the *emmeans* package (Lenth, 2020). An α-level of 0.05 was adopted throughout.

The first model (Model 1) was built to assess (1) if the two speaker groups (Bilingual vs. Control) differed in terms of perceived nativeness, and (2) if the two rater groups (BIL vs. ML) differed in terms of their perception of nativeness of monolingual and bilingual L1 speech. Model 1 included *rating score* as the dependent variable, *Rater_Group* (two levels: BIL, ML), *Speaker_Group* (two levels: Bilingual, Control) and an interaction between the two as independent variables, and *Rater*, *Speaker* and *Stimuli* as random factors. The model contained *Rater*-, *Speaker*-, and *Stimuli*-specific intercepts and by-*Rater* random slopes for *Speaker_Group*, by-*Speaker* random slopes for *Rater_Group*, and by-*Stimuli*-specific random slopes for *Rater_Group*. The second model (Model 2) aimed to test if the two listener groups (BIL vs. ML) differed in terms of their perception of nativeness of the individual bilingual speakers to determine if some of the bilinguals were perceived to be more or less native by the two listener groups, respectively. In this model, rating score was included as the dependent variable, and *Rater_Group* (two levels: BIL, ML), *Speaker* (nine levels: one for each of the speakers), and an interaction between *Rater_Group* and *Speaker* as independent variables. *Rater* and *Stimuli* were introduced as random factors, with random slopes for *Speaker* (by *Rater*) and *Rater_Group* (by *Stimuli*).

## Results

Interrater reliability was assessed for each of the two listener groups by calculating Cronbach’s alpha coefficient. Inter-rater reliability was high in both the BIL (α = 0.84) and the ML (α = 0.89) listener group.

**FIGURE 1:**
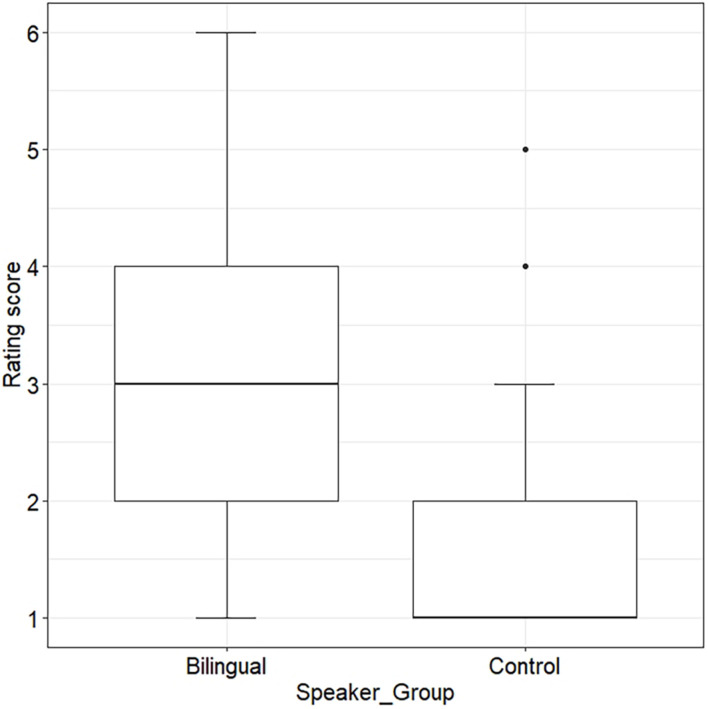
Distribution of nativeness rating scores from 1 = *certain of native speaker status* to 6 = *certain of non-native speaker status* by speaker group (Bilingual vs. Control).

Figure 1 shows the rating scores obtained for the bilingual speaker group and the AG control group. On the six-point scale, ranging from 1 = *certain of native speaker status* to 6 = *certain of non-native speaker status*, monolingual control speakers received a median score of 1.0 (*min-max* = 1.0–5.0) and bilingual speakers were rated with a median score of 3.0 (*min-max* = 1.0–6.0), which suggests that the bilinguals were perceived to sound overall less native in their L1 compared to monolingual AG speakers. Model 1 (see section “Statistical Analysis”) showed a main effect for *Speaker_Group*, χ^2^[1] = 7.38, *p* < 0.001, confirming that the two speaker groups significantly differed in terms of the rating scores they obtained, β = 4.77, *SE* = 0.866, *z* = 5.51, and *p* < 0.001.

**FIGURE 2:**
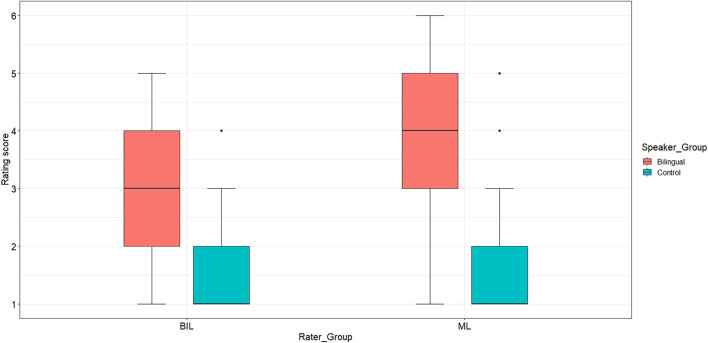
Distribution of nativeness rating scores from 1 = *certain of native speaker status* to 6 = *certain of non-native speaker status* by rater group [Bilingual (BIL) raters vs. Monolingual (ML) raters].

Figure 2 displays the rating scores the two rater groups (BIL vs. ML) assigned to the bilingual and monolingual speaker group, respectively. ML listeners were overall more likely to rate the bilinguals’ L1 pronunciation as sounding non-native (*Mdn* = 4.0, *min-max* = 1.0–6.0) compared to the BIL listeners (*Mdn* = 3.0, *min-max* = 1.0–5.0). At the same time, both rater groups judged the bilingual speakers to sound overall less native than the monolingual controls (*Mdn* = 1.0, min-max = 1.0–4.0). The statistical analysis (Model 1) revealed significant main effects for *Rater*_*Group*, χ^2^[1] = 5.68, *p* < 0.001, *Speaker*_*Group*, χ^2^[1] = 7.38, *p* < 0.001, and a significant interaction between the two, χ^2^[1] = 17.75, *p* < 0.001. Pairwise comparisons showed significant differences between the listener groups in terms of the nativeness ratings assigned to the bilingual speakers, β = −2.85, *SE* = 0.54, *z* = −5.28, and *p* < 0.001, that is, BIL listeners were less likely to rate bilingual speakers as non-native compared to ML listeners. By contrast, no significant differences between the rater groups were identified concerning their judgments of the control speakers (*p* = 0.21), i.e., both BIL and ML listeners perceived the monolingual controls to sound equally native.

**FIGURE 3:**
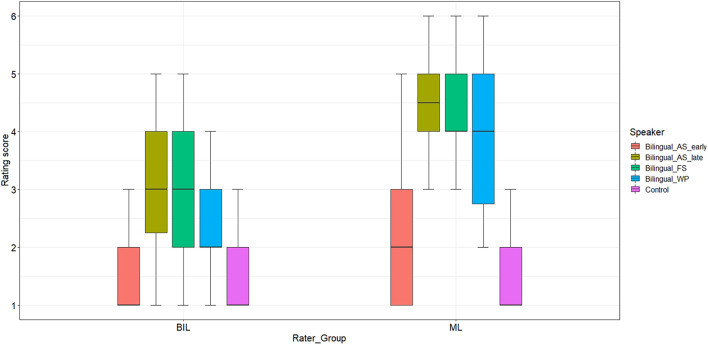
Distribution of nativeness rating scores from 1 = *certain of native speaker status* to 6 = *certain of non-native speaker status* by rater group [Bilingual (BIL) raters vs. Monolingual (ML) raters] and individual speakers.

Figure 3 shows the rating scores the two rater groups assigned to the individual bilingual speakers and the control group. The ML listener group rated the speech samples produced by AS_late with a median score of 4.5 (*min-max* = 1.0–6.0), and speakers FS and WP both received a median score of 4.0 (*min-max* = 2.0–6.0). By contrast, the three bilinguals received a considerably lower rating score from the BIL rater group (AS_late: *Mdn* = 3.0, *min-max* = 1.0–5.0; FS: *Mdn* = 3.0, *min-max* = 1.0–5.0; and WP: *Mdn* = 2.0, *min-max* = 1.0–4.0). Noticeably, both the BIL and the ML listeners judged speaker WP to sound more native compared to speakers AS_late and FS, but less native than the monolingual control group. It can be further observed that the speech samples produced by AS_early were rated to sound as native as the control speaker samples by the BIL listeners (*Mdn* = 1.0, *min-max* = 1.0–4.0) while the ML listeners judged AS_early to sound slightly less native (*Mdn* = 2.0, *min-max* = 1.0–5.0) than the monolingual controls (*Mdn* = 1.0, *min-max* = 1.0–5.0).

Model 2, including *Rater*_*Group*, *Speaker* and an interaction between the two as fixed factors, and *Rater* and *Stimuli* as random factors, showed significant main effects for *Rater*_*Group*, χ^2^[1] = 23.15, *p* < 0.001, and *Speaker*, χ^2^[8] = 405.88, *p* < 0.001, as well as a significant interaction between *Rater_Group* and *Speaker*, χ^2^[8] = 24.82, *p* < 0.001. *Post hoc* pairwise comparisons revealed that the rating differences between the two listener groups were highly significant for AS_early, β = −3.07, *SE* = 0.79, *z* = −3.87, *p* < 0.001, AS_late, β = −3.81, *SE* = 0.68, *z* = −5.6, *p* < 0.001, FS, β = −3.45, *SE* = 0.69, *z* = −5.07, *p* < 0.001, and WP, β = −3.21, *SE* = 0.76, *z* = −4.22, *p* < 0.001, again confirming that BIL raters were less likely than ML raters to judge the bilinguals’ L1 speech as non-native. While the rating differences between AS_late and FS were not shown to be significant in both the BIL (*p* = 1.0) and the ML (*p* = 0.99) rater group, the rating differences between WP and AS_late, BIL: β = 1.83, *SE* = 0.46, *z* = 3.96, and *p* < 0.001, ML: β = 2.44, *SE* = 0.51, *z* = 12.13, and *p* < 0.001, and WP and FS, BIL: β = 1.91, *SE* = 0.44, *z* = 4.33, *p* < 0.001, ML: β = 2.2, *SE* = 0.4.7, *z* = 4.66, and *p* < 0.001, turned out to be significant for both rater groups. That is, in both rater groups, speaker WP was perceived to sound more native in his L1 than AS_late and FS, but still less native than the monolingual control speakers, BIL: β = −3.96, *SE* = 0.23, *z* = −17.2, and *p* < 0.001; ML: β = −5.72, *SE* = 0.25, *z* = −22.41, and *p* < 0.001. The observation that the two rater groups differed in terms of their judgments of AS_early (see Figure 3) was also confirmed in the statistical analysis. ML listeners judged AS_early to sound less native in his L1 compared to the monolingual controls, β = −2.59, *SE* = 0.27, *z* = −9.71, and *p* < 0.001, while BIL listeners perceived his L1 pronunciation to be as native as the control speakers’ AG pronunciation, that is, the rating differences between AS_early and the control group were not shown to be significant (*p* = 0.26). In addition, no significant differences were identified in terms of the ML and BIL listeners’ rating scores assigned to the individual monolingual control speakers.

## Discussion

### Perceived Nativeness of First Language Pronunciation

The present study aimed to (1) determine if long-term L2-immersed bilinguals are perceived to sound less native in their L1 when being compared to monolingual AG controls speakers, and (2) identify if and to what extent listeners’ linguistic background (monolingual vs. bilingual) affects their perception of nativeness.

With regard to the first research aim, the nativeness rating experiment showed that, overall, bilingual speakers who started acquiring their L2 English as adults and who have been long-term immersed in an English-speaking country for several decades were, in fact, perceived to sound less native in their native AG pronunciation compared to monolingual AG control speakers. The observation that long-term L2 learning experience in an immersion setting may lead to a non-native L1 accent is in line with the findings of previous L1 accent rating studies (Sancier and Fowler, 1997; De Leeuw et al., 2010; Hopp and Schmid, 2013; Schmid and Hopp, 2014; Bergmann et al., 2016; Mayr et al., 2020). Taken together, these studies undermine the assumption that an individual’s L1 system—once fully matured—remains impermeable and stable throughout the lifespan and is not prone to be altered by a late-acquired L2 system (e.g., Lado, 1957; Lenneberg, 1967; Scovel, 1969). Instead, the present and previous findings show that a bilingual’s L1 system can indeed be modified resulting from a dynamic interaction between the two linguistic systems (see e.g., de Bot et al., 2007; de Bot and Larsen-Freeman, 2011; de Leeuw et al., 2012a), which might, in turn, entail that speakers’ L1 pronunciation is perceived to sound less native when being compared to monolingual controls.

Alongside the observation that the L1 speech produced by bilingual speakers was perceived to sound considerably less native compared to monolingual speech, the present study revealed significant effects of listeners’ linguistic background on their nativeness judgments. That is, native AG listeners who were actively learning L2 English and using their L2 in different contexts were more lenient in their judgments compared to (quasi-)monolingual AG listeners who were overall more likely to judge bilingual L1 speech to sound non-native. These findings add a further dimension to the observation that “[f]oreign accent is not only the way the learners produce the L2, but also what the native speakers of the target language *perceive* as such” (Reinisch, 2005, p. 82; our emphasis). As the present findings reveal, the same applies to a speaker’s L1, which is not only influenced by speaker-related pronunciation features, but the extent to which a speaker is perceived to be a native speaker of their L1 also depends on who is listening. As addressed in Section “Listener Variables,” previous studies examining potential effects of listener variables on the perception of L1 (Schmid and Hopp, 2014) and L2 (e.g., Thompson, 1991; Flege and Fletcher, 1992; Cunningham-Andersson, 1997; Kennedy and Trofimovich, 2008; Eger and Reinisch, 2019) speech provide rather inconclusive findings as to whether listeners’ linguistic background and experience influence their perception of nativeness and accentedness. Some studies report an effect of listener experience on their judgments of nativeness, showing that linguistically experienced listeners are more tolerant toward non-native L2 speech compared to listeners without linguistic experience (Thompson, 1991). In the context of the present investigation, similar observations were made with regard to listeners’ perception of L1 speech. Others, by contrast, have demonstrated that experienced listeners are more sensitive to accented speech and, therefore, are more likely to judge a speaker as sounding non-native (Eger and Reinisch, 2019), and still others have concluded that listener experience does not have a significant effect on nativeness judgments (Flege and Fletcher, 1992; Kennedy and Trofimovich, 2008). One reason for these—to some extent—contradictory findings might be that these studies use different criteria for classifying experienced vs. inexperienced listeners, ranging from self-reported L2 proficiency (e.g., Eger and Reinisch, 2019), being fluent L2 speakers with frequent non-native speaker contact (e.g., Thompson, 1991), to being familiar with non-native L2 speech, but not necessarily actively using an L2 (e.g., Flege and Fletcher, 1992; Major, 2007). It might be that being merely exposed to non-native speech without using an L2 in different communicative contexts is not sufficient to increase listeners’ tolerance for accented speech. By contrast, being frequently exposed to non-native speech and using an L2 on a regular basis—as the listeners in Thompson (1991) and in the present study—makes listeners more lenient and perhaps less sensitive toward accented speech. In addition, it must be taken into consideration that linguistic experience is, of course, not the only listener-dependent variable which might affect judgments of native vs. non-native speech. As pointed out by Munro et al. (2006), listener prejudices toward non-native accents may decrease their tolerance for accented speech while positive attitudes toward a particular accent are likely to make listeners being more lenient in their judgments of (non-)native speech (e.g., Beinhoff, 2013; Kraut and Wulff, 2013; Kraut, 2014). The extent to which additional listener variables, such as language attitudes, are correlated with listener experience and their perception of L1 pronunciation certainly offers a fruitful incentive for future research.

A third observation which has been made based on the results obtained in the nativeness rating study was that not all bilinguals were perceived to sound (non-)native to the same degree. For instance, both ML and BIL listeners perceived the bilingual speaker WP to sound more native in his L1 compared to the other two bilinguals, but still less native than the monolingual controls. The observation that bilingual speakers may differ in the degree of perceived nativeness—to the extent that some speakers are in fact not perceived differently from monolingual speakers (see section “First Language Phonetic Attrition”)—has been made in previous L1 accent rating studies (e.g., Major, 1992; De Leeuw et al., 2010; Bergmann et al., 2016), which mirror the findings of studies focusing on perceived L2 accent (e.g., Bongaerts, 1999; Moyer, 1999). These studies show that even in groups of bilinguals who were carefully matched across a variety of factors, including age of L2 acquisition, proficiency, and length of residence in an L2 speaking country, speakers were not necessarily rated to sound equally (non-)native in their L2 speech. Similarly, some bilinguals might be perceived as relatively more or less native in their L1 pronunciation compared to other bilinguals. Alongside the reasons outlined in Section “First Language Phonetic Attrition,” the observation that speakers may differ in the degree of perceived nativeness might account for an influence of additional internal and external factors, such as language learning aptitude or quantity and quality of target language input, which have been previously shown to affect the degree of non-native accent in L2 learning contexts (see Piske et al., 2001, for an overview). Only few investigations so far have systematically examined the role of such variables in the context of perceived nativeness of L1 speech (e.g., Hopp and Schmid, 2013). Hopp and Schmid (2013), for instance, found an effect of language aptitude on perceived foreign accent in the L1 speech of German-English and German-Dutch bilinguals, that is, speakers with high levels of language aptitude were, overall, judged to sound more native compared to speakers with comparatively lower levels of language aptitude, suggesting that language aptitude protects to some extent against L1 attrition effects. It should be noted though that Hopp and Schmid (2013) did not directly test language aptitude, but used a language proficiency test as an indirect measure of aptitude. Other factors, including frequency of language use and language attitudes, were not found to have a predictive effect on L1 nativeness ratings. Despite the fact that further systematic research is certainly necessary to get a better understanding of the role these factors effectively play when it comes to the perception of nativeness of L1 pronunciation and speakers’ ability to retain a native accent in their L1, the observation made in the present study that not all bilinguals were perceived to sound equally non-native in their L1 speech might be explained against the background of additional influencing factors.

Interestingly, significant differences in the perception of nativeness were also observed when comparing the ratings obtained for the early (1970s) and late (2010s) speech produced by the bilingual AS. His early L1 pronunciation was judged to sound more native than his late pronunciation, but at the same time overall less native than the monolinguals’ pronunciation—at least when considering the judgments made by the ML listeners. These findings show that the extent to which a speaker is perceived to sound native in their L1 might change over time in response to L2 learning experience and being immersed in an L2-setting. A previously conducted acoustic-phonetic investigation of AS’s L1 vowels and plosives has, in fact, revealed a similar trend, namely that—in the course of L2-immersion—some of his L1 vowel and plosive targets have become less native-like, i.e., have moved away from native production norms (Kornder and Mennen, 2021). A reason why AS’s early pronunciation was rated to sound less native compared to the monolinguals’ AG pronunciation in the present investigation might be that the speech recordings representing his early pronunciation were made in the late 1970s, that is, approximately 10 years after he had migrated to the United States. Earlier recordings were not available at the time the present study was conducted. Hence, after a decade of L2 immersion, it can be assumed that AS had already gained quite some L2 learning experience which may have led to changes in his L1 pronunciation. As such, the present findings provide further evidence for the plasticity of a bilingual’s L1 pronunciation system and show that a mature L1 is not robust over the lifespan. Given, however, that these observations were made for a single speaker only, future empirical studies need to examine such changes over time more closely, focusing on larger populations of bilingual speakers.

As outlined above, the observation that monolingual and bilingual listeners differ in their judgments of L1 pronunciation has been interpreted in the context of *listener tolerance*. We argue that speaking an additional language on a regular basis and use the L2 in different contexts increases listeners’ tolerance for L1 speech which might diverge from expected L1 pronunciation norms. Conversely, listeners who lack frequent and regular exposure to an L2 seem to be more sensitive toward non-native pronunciation patterns, which makes them being less lenient in their nativeness judgments. However, an alternative explanation is that the use of an additional language has led to a perceptual restructuring of the L1. As mentioned in Section “First Language Phonetic Attrition,” cross-linguistic interactions between a speaker’s L1 and L2 linguistic system are not restricted to highly experienced and long-term immersed bilinguals, but have been observed to occur at different stages of bilingualism, that is, in both beginner L2 learners (e.g., Chang, 2012, 2013; Kartushina et al., 2016b) and highly experienced L2-immersed bilinguals (e.g., Mayr et al., 2012; Stoehr et al., 2017; de Leeuw, 2019; Kornder and Mennen, 2021). These L1–L2 interactions do not only lead to acoustic-phonetic changes in a bilingual’s L1 pronunciation system, but might also affect their ability to perceive a non-native accent in their L1 (see Major, 2010, for a discussion). In the context of the SLM; (Flege, 1995; Flege and Bohn, 2021), it is argued that a bilingual’s L1 and L2 sound system mutually influence each other, which has been shown to lead to a restructuring of speakers’ L1 phonetic categories in the direction of the L2 (e.g., Flege and Hillenbrand, 1984; Flege, 1987b, 1991; Mack, 1990). Moreover, the SLM predicts that “a strong bidirectional connection exists between production and perception” (Flege and Bohn, 2021, p. 29), that is, sound perception and production are considered to be interrelated. If we assume that L1-modifications resulting from L2 learning experience do not only influence speech production, but also affect speech perception, then it might be argued that the bilingual listeners in the present study have experienced underlying changes in their perception of L1 speech, triggered by their own bilingual background and L2 learning experience. Despite the fact that most studies examining phenomena of L1–L2 interactions in late sequential bilinguals focus on speech production either at the segmental level (e.g., Mayr et al., 2012; Stoehr et al., 2017; Kornder and Mennen, 2021) or at the level of global accent (e.g., De Leeuw et al., 2010; Bergmann et al., 2016; Mayr et al., 2020), some investigations set out to explore to what extent L1 speech perception in adult listeners is influenced by the L2 (Caramazza et al., 1973; Flege et al., 1999; Major, 2010; Alcorn and Smiljanic, 2017; Cabrelli Amaro, 2017; Carlson, 2018; Cabrelli et al., 2019). These studies either examine bilinguals’ ability to discriminate native vs. non-native L1 pronunciation (Major, 2010), or assess bilingual listeners’ perceptions of individual L1 segments, sound contrasts, or suprasegmental features (e.g., Caramazza et al., 1973; Flege et al., 1999; Alcorn and Smiljanic, 2017; Cabrelli Amaro, 2017; Carlson, 2018; Cabrelli et al., 2019). Some of these investigations provide evidence for a partial perceptual restructuring of the L1, showing that a late-acquired L2 does not only influence L1 production, but might also have an effect on L1 perception abilities (see e.g., Cabrelli et al., 2019, for Portuguese-English; Carlson, 2018, for Spanish-English; Celata and Cancila, 2010, for Lucchese-English). In the light of these findings, we might expect a restructuring of L1 perception also in the present study. That is, bilingual listeners’ perception of L1 pronunciation might have been altered resulting from an interaction between their L1 and L2 system, which made them judge the nativeness of L1 pronunciation differently from listeners who do not speak an additional language. As a result, one and the same speaker might be perceived differently, depending on who is listening. This certainly entails methodological consequences for studies examining perceived global accent in that potential raters need to be carefully screened for their linguistic background and experience, acknowledging that these variables might influence their perception of L1 and presumably also L2 speech. Moreover, the observation that the extent to which a speaker is perceived to be a native speaker is influenced by a listener’s own language background is relevant with regard to the concept of the native speaker, as will be further discussed below.

A last aspect which needs to be addressed when interpreting the findings obtained in the present study are potential range effects, which relate to the ratio of bilingual (or non-native) and monolingual speech samples represented in a rating task (see Flege and Fletcher, 1992; Schmid and Hopp, 2014). As Flege and Fletcher (1992) observed, listeners are more likely to judge non-native speakers as sounding foreign in rating tasks including a higher number of native control samples compared to non-native samples. In the present study, such range effects were reduced to some extent by including a higher number of bilingual samples (*N* = 18) and a comparatively lower number of monolingual samples (*N* = 10). Still, it should be taken into consideration that the bilingual samples were produced by three individual speakers only, which is—compared to previous accent rating studies (e.g., De Leeuw et al., 2010; Hopp and Schmid, 2013; Bergmann et al., 2016; Mayr et al., 2020)—a relatively small number. This may have influenced the reported results in that the three bilingual speakers might have stood out from the overall small number of speakers and were therefore more likely to be judged as sounding non-native in their L1 pronunciation.

### Challenging “the Native Speaker”

Taken together, the findings of the present investigation do not only make an empirical contribution to the field of bilingual speech development in that they provide evidence for the malleability of the native language system and show that speakers might be perceived as non-native speakers of their L1, but they also add a new perspective to the broader notion of nativeness and the concept of the native speaker (e.g., Davies, 2003, 2004). Discussions related to the phenomenon of the native speaker have been evolving in various subfields of linguistics in the past decades, including, for example, sociolinguistics (e.g., Coulmas, 1981), language teaching (e.g., Leung et al., 1997; Cook, 1999), and multilingualism/bilingualism (e.g., Dostert, 2009; Rothman and Treffers-Daller, 2014). In its original, most basic definition, *the native speaker* has been described as a standard setter, portraying the embodiment of “true” language (see Davies, 2004), against which speakers are evaluated and judged. Evaluations and judgments of this kind do, in many cases, lead to accent-based discrimination, which so far has been predominantly considered in the context of non-native L2 speech (see e.g., Lippi-Green, 1997). But what happens if an individual is perceived to have a non-native accent in both their second and their first language? Despite the observation that there are non-native accents which listeners may consider more “acceptable” than others—including, for instance, prestigious European accents (Munro and Derwing, 2009)—speaking with a non-native accent in both the L1 and the L2 can entail serious personal and psycho-social consequences for individuals. These range from difficulties to access the job market (Munro, 2003), suffering from unequal payment (Dávila et al., 1993) to experiencing a profound strain on one’s sense of belonging (Lippi-Green, 1997). Accent-based discrimination and stereotyping are inherently connected to the common belief that something like “true” nativeness—in the sense of accent-free speech—exists. However, as the findings of the present investigation illustrate, there is no single stable criterion based on which it would be possible to define *true* nativeness or the native speaker. If and to what extent speakers are perceived as native speakers depends on a variety of factors on behalf of both speakers and listeners. That is, the same speaker might be perceived differently by different listeners, whose perceptions are shaped and influenced by their own linguistic backgrounds and presumably by additional variables, such as their attitudes toward specific languages and accents (e.g., Beinhoff, 2013; Kraut and Wulff, 2013; Kraut, 2014). Furthermore, the present findings reveal that a speaker might “lose” their status as native speaker over time, which further supports the view that the native speaker—in the sense of reflecting a stable and coherent concept—as such might not exist, particularly in the context of bilingualism. As pointed out by Cook (1999; see also Cook, 2003), bilinguals differ from monolingual speakers not only in their knowledge of an additional language, but also in terms of their cognitive processes, which essentially supports a holistic perspective on bilingualism (Grosjean, 1989). Hence, evaluating bilingual speech against monolingual standards and regarding monolingualism as the “benchmark of true nativeness,” as Rothman and Treffers-Daller (2014, p. 93) put it, is rather misleading and does not properly reflect linguistic reality—considering that we live in a world where more than half of the population speaks more than one language (e.g., Aitchison, 1994; Grosjean, 2010). The observations made in the present study that bilingual speakers might no longer be perceived as native speakers of their L1 and that the perception of nativeness is strongly influenced by listeners’ personal linguistic experience point to the need to reassess the static and idealized image of nativeness and to acknowledge the inherently dynamic nature of language and speech development.

## Data Availability Statement

The raw data supporting the conclusions of this article will be made available by the authors, without undue reservation.

## Ethics Statement

The studies involving human participants were reviewed and approved by the Ethics Committee of the University of Graz. The patients/participants provided their written informed consent to participate in this study.

## Author Contributions

LK designed and conducted the experiment, analyzed and visualized the data, and wrote the manuscript. IM helped with the experiment design, wrote, reviewed, and edited the manuscript. Both authors have read and approved the final version of the manuscript.

## Conflict of Interest

The authors declare that the research was conducted in the absence of any commercial or financial relationships that could be construed as a potential conflict of interest.

## Publisher’s Note

All claims expressed in this article are solely those of the authors and do not necessarily represent those of their affiliated organizations, or those of the publisher, the editors and the reviewers. Any product that may be evaluated in this article, or claim that may be made by its manufacturer, is not guaranteed or endorsed by the publisher.

## Footnotes

1 Raters were not matched according to age or gender given that there is no empirical evidence that these variables significantly affect accent ratings (see Schmid and Hopp, 2014).

2 It is difficult—if not impossible—to find monolingual speakers in Austria who do not have at least some very basic knowledge of English (or other foreign languages) given that English has been a compulsory subject in Austrian schools since the mid–twentieth century (see de Cillia and Krumm, 2010).

3 One reason for this might be that only some of the subjects included in the BIL group, but all ML subjects, had a (part-time) job given that they were university students at the time the present study was conducted.

4 All instructions and questions were presented to the listeners in German. On the original rating questionnaire, each listener was asked (1) Hat dieser Sprecher Deutsch als Muttersprache? (Is this speaker a native German speaker?), and (2) Wie sicher sind Sie sich? (How certain are you of this?), with three answer options (sicher/certain; relativ sicher/relatively certain; and unsicher/uncertain).
