# Supplementary material for: Listeners’ Linguistic Experience Affects the Degree of Perceived Nativeness of First Language Pronunciation
Source: Front Psychol. 2021 Oct 8;12:717615. doi: 10.3389/fpsyg.2021.717615 (PMC8531519; doi:10.3389/fpsyg.2021.717615)
Supplement: Supplementary file 1 [file Table_1.DOCX]

Supplementary Table 1. Overview of speech samples used in the rating experiment.

| Speaker | Transcript | Duration (in sec.) |
| --- | --- | --- |
| Sp1_Control | Naja ich hätt einmal gesagt so im Durchschnitt mit der richtigen Frequenz fürs Herz | 4.71 |
| Sp1_Control | Es gibt ja auch immer wieder von Freitag auf Samstag ein Nachtfischen | 3.65 |
| Sp2_Control | Da war jetzt einer dabei die Kugel ist auffigangen jetzt geht sie obi | 3.46 |
| Sp2_Control | Der hat das Fischerl jetzt genommen | 2.6 |
| Sp3_Control | Einen modernen Rapper oder Schlager der sagt zweihundert Mal das gleiche hintereinander | 5.39 |
| Sp3_Control | Das ist sehr häufig noch immer das hat man heut noch immer ich geh ume | 4.04 |
| Sp4_Control | Er fühlt sich sehr schuldig gegenüber der Kinder und seiner Frau | 4.71 |
| Sp4_Control | Und da hab ich ihn gefragt darf ich auch einmal probieren ja bitte komm her da | 4.72 |
| Sp5_Control | Natürlich schaut das jetzt alles ganz anders aus das ist fünfzig Jahre inzwischen her | 4.98 |
| Sp5_Control | Dieser Stein ist draußen gestanden beim Freizeitpark | 3.21 |
| AS_early_1 | Ich wollte irgendwie ein Mittel finden dass ich das erreich | 4.27 |
| AS_early_2 | Und am sechsundzwanzigsten April haben wir geheiratet | 3.37 |
| AS_early_3 | Wie gesagt früher oder später wird das schon passieren | 3.47 |
| AS_late_1 | Das Wichtigste ist dass man immer viel Spaß dabei hat | 3.41 |
| AS_late_2 | Die strenge Erziehung war sehr wichtig | 2.69 |
| AS_late_3 | Ich fahr ungefähr eine Stunde mit dem Rad | 1.95 |
| FS_1 | Die richtigen Eigenschaften hat dann kann sehr viel passieren | 3.68 |
| FS_2 | Sie sind auch so begabt dass sie sich selbst zerstören können nicht | 4.57 |
| FS_3 | Wir haben jetzt schon über acht Milliarden Menschen auf der Erde | 4.01 |
| FS_4 | Und ich kann mich noch gut erinnern sagen wir vor vierzig fünfzig Jahren | 4.16 |
| FS_5 | Darum dass man gewisse Dinge weitergibt ja | 2.55 |
| FS_6 | Habe ich keinen Stress dass ich das versäume | 2.68 |
| WP_1 | Und da haben wir ein Drittel von unseren Kunden verloren | 3.52 |
| WP_2 | Zum Beispiel ich habe vor neunzehnhundertneunzig eine Brauerei aufgemacht | 4.5 |
| WP_3 | Und das Gasthaus ist wirklich wunderbar gegangen | 3.28 |
| WP_4 | Und ich mach nur die scharfen Grenzen die sie nicht überschreiten können | 5.21 |
| WP_5 | Am Ende kann man das nicht wirklich kontrollieren | 2.54 |
| WP_6 | Eine Currywurst ich weiß das schon hoffentlich schmeckts besser als wie's ausschaut | 4.58 |
